# Dual pathway activation in wound repair: An in vitro study of betanin and theaflavin on periodontal ligament fibroblasts

**DOI:** 10.1016/j.jobcr.2025.10.020

**Published:** 2025-11-11

**Authors:** Arwa Alihusain Kapasi, Taniya Mary Martin, Meenakshi Sundaram Kishore Kumar, Jayalakshmi Somasundaram, Kommireddy Vaishnavi

**Affiliations:** a White Lab - Material Research Centre, Department of Conservative Dentistry and Endodontics, Saveetha Institute of Medical and Technical Sciences (SIMATS), Saveetha University, Chennai, 600077, Tamil Nadu, India; b Zebrafish Facility, Department of Anatomy, Saveetha Institute of Medical and Technical Sciences (SIMATS), Saveetha University, Chennai, 600077, Tamil Nadu, India

**Keywords:** Betanin, Theaflavin, Periodontal ligament fibroblasts, Wound healing, In vitro study, Angiogenesis, Anti-inflammatory

## Abstract

**Background:**

Wound healing is a highly coordinated biological process involving inflammation resolution and neovascularization. Dysregulation of these processes can result in chronic wounds, especially in inflammatory or oxidative stress–rich environments. There is growing interest in natural compounds that can simultaneously promote angiogenesis and suppress inflammation to enhance wound healing outcomes.

**Objective:**

This study aims to evaluate the synergistic wound-healing effects of betanin and theaflavin in vitro by assessing their ability to enhance cell viability, migration, and gene expression related to inflammation and angiogenesis in human periodontal ligament (PDL) fibroblasts.

**Methods:**

PDL fibroblasts were treated with betanin, theaflavin, and their combinations at two concentrations (*viz.,* 1, 5, 10, 25, 50, 75, 100, 125 μg/mL) were preliminary screened by 3-(4,5-dimethylthiazol-2-yl)-2,5-diphenyltetrazolium bromide (MTT) assay and based on the IC_50_ values, further tested with two concentrations (10 μg/mL and 25 μg/mL, individually and in combination). The scratch assay was employed to evaluate fibroblast migration. To assess molecular changes, qRT-PCR was performed to quantify expression levels of VEGF-A, HIF-1α, NF-κB, IκBα, and IL-10.

**Results:**

Combination treatment significantly enhanced cell viability and wound closure compared to individual treatments and controls. Gene expression analysis showed increased expression of VEGF-A and HIF-1α, indicating improved angiogenic potential. Concurrently, NF-κB expression was reduced, while IκBα and IL-10 were upregulated, indicating anti-inflammatory activity.

**Conclusion:**

The combined use of betanin and theaflavin enhances in vitro wound healing by promoting angiogenesis and modulating inflammatory responses. This integrative strategy may offer a promising natural therapeutic approach for managing tissue repair in oral and dermal wounds.

## 1 Introduction

Wound healing is a complex and dynamic process that involves the activation and resolution of multiple cellular events aimed at restoring tissue integrity.^1^ The process is typically categorized into four interrelated and overlapping stages: hemostasis, inflammation, proliferation, and remodeling. Each phase is governed by a tightly regulated network of signaling molecules, cytokines, growth factors and extracellular matrix components. When these stages are interrupted or dysregulated due to factors such as infection, oxidative stress, or metabolic disease healing becomes delayed or dysfunctional, leading to chronic wound formation.^2^ Two biological processes, inflammation and angiogenesis, are particularly critical to successful wound healing. In the early phases of healing, inflammation plays a defensive role, eliminating pathogens and necrotic debris while signaling other cells to migrate to the wound site. However, if inflammation becomes prolonged, it can hinder healing by promoting tissue destruction, fibrosis, and oxidative damage. Thus, timely resolution of inflammation is essential for transitioning into the proliferative phase, where new tissue formation occurs.^3^^,^^4^

Simultaneously, in angiogenesis the formation of new blood vessels from pre-existing vasculature. It is essential for delivering nutrients, oxygen, and cellular components to support regeneration. It plays a pivotal role during the proliferative phase by enabling granulation tissue formation and re-epithelialization. Angiogenesis also supports fibroblast function, collagen deposition, and the recruitment of progenitor cells. Consequently, effective wound healing requires a therapeutic approach that can both suppress chronic inflammation and stimulate angiogenic pathways.^5^

In recent years, there has been a shift toward exploring plant derives bioactive compounds for wound management. Natural molecules offer several advantages, including multi-target activity, low cytotoxicity, and antioxidant potential.^6^ Among the most promising candidates, betanin and theaflavin are well-known for their distinct yet complementary biological effects. Betanin is a water-soluble red-violet pigment extracted from beetroot. It is known for its strong antioxidant capacity, which enables it to neutralize reactive oxygen species (ROS) that are abundant in the wound environment. ROS, while playing a signaling role in low concentrations, can cause significant cellular damage and delay healing when present in excess.^7^ Betanin helps to restore redox balance and protects cells from oxidative stress-induced injury. Betanin has been demonstrated to interfere with pro-inflammatory signaling pathways and alter important inflammatory mediators in addition to its antioxidant properties. It also promotes angiogenesis by enhancing the expression of vascular growth factors, making it an ideal candidate for tissue regeneration strategies.^8^

Theaflavin, on the other hand, is a polyphenolic compound formed during the fermentation of black tea leaves. It has received attention for its ability to modulate inflammation through the downregulation of inflammatory cytokines and suppression of specific transcriptional regulators involved in the immune response. Theaflavin also possesses endothelial-protective effects, helping to maintain vascular integrity and function under stress. Moreover, its antioxidant profile complements that of betanin, enabling the reduction of oxidative damage in wounded tissues.^9^^,^^10^ While both compounds have individually demonstrated therapeutic potential in various preclinical settings, their combined use in the context of wound healing has not been extensively evaluated. The rationale for combining betanin and theaflavin lies in their overlapping antioxidant and anti-inflammatory properties, along with their distinct capacities to influence angiogenesis and cell survival. Integrating both agents could results in enhanced wound healing effects through synergistic modulation of key cellular and molecular processes.^9^^,^^10^

The present study investigates the combined application of betanin and theaflavin using an in vitro model of wound healing. Human periodontal ligament (PDL) fibroblasts were selected for this investigation due to their relevance in connective tissue repair, especially in the oral cavity. These fibroblasts support matrix remodeling, angiogenesis, and epithelial regeneration in addition to being essential for preserving periodontal integrity.^11^

All treatments were performed under standardized cell culture conditions, and each experiment was repeated in triplicate to ensure statistical reliability^12^^,^^13^^,16.^

We hypothesize that targeting inflammation and angiogenesis simultaneously by betanin and theaflavin might offer a novel and biocompatible approach for accelerating tissue regeneration in dental, periodontal, and possibly dermal applications^14^ than their individual applications.

## 2 Materials and methods

### 2.1 Cell culture and reagents

Human periodontal ligaments (PDL) fibroblasts were selected as the in vitro model due to their critical role in soft tissue repair and wound healing processes. To ensure optimal growth and preserve sterility, cells were obtained from a certified cell repository and grown in Dulbecco's Modified Eagle Medium (DMEM, Gibco, USA), supplemented with 10 % fetal bovine serum (FBS, Gibco, USA) and 1 % penicillin-streptomycin (Gibco, USA). The cultures were kept in a humidified incubator with 5 % CO_2_ at 37 °C under normal conditions. To maintain experimental consistency and prevent phenotypic drift brought on by extended passaging, only cells from passages three through six were employed. Commercial vendors provided the highly purified powdered forms of bethanin and theaflavin. Stock solutions were prepared by dissolving the compounds in dimethyl sulfoxide (DMSO, Gibco, USA) and stored at −20 °C in amber vials to prevent degradation. To obtain the required treatment concentrations, working solutions were freshly diluted in culture medium before each experiment. The final concentration of DMSO in all experimental groups was maintained below 0.1 % to eliminate any solvent-induced cytotoxic effects and to serve as a vehicle control benchmark.^14^

### 2.2 Stock solution preparation

Stock solutions of betanin and theaflavin were prepared to ensure accurate dosing and stability throughout the experiment period. To make concentrated stock solutions, both chemicals were first dissolved in dimethyl sulfoxide (DMSO) after being obtained in high-purity powdered form. In order to guarantee full solubilization, betanin and theaflavin were each dissolved to a concentration of 10 mg/mL with little vortexing. To avoid light-induced deterioration, the stock solutions were aliquoted into amber microcentrifuge tubes and kept at −20 °C until they were needed again. To reach final treatment doses of 10 μg/mL, either separately or in combination, fresh working dilutions were made from the stock for each experiment by diluting it into culture media that had been preheated. The final concentration of DMSO in all experimental groups, including the vehicle control, was maintained at or below 0.1 % to minimize solvent-induced cytotoxicity.^15^^,^^16^

### 2.3 Cell culture treatment groups

Cytotoxicity of the compounds were analysied using 3-(4,5-di methyl thiazol-2-yl)-2,5-diphenyltetrazolium bromide (MTT, Sigma, USA) assay. In preliminary analysis, eight concentrations (*viz.,* 1, 5, 10, 25, 50, 75, 100, 125 μg/mL) were tested against PDL cells at 24h duration. Based on these results, For the experimental design, human periodontal ligament (PDL) fibroblasts were divided into six distinct groups to evaluate the individual and combined effects of betanin (Sigma, USA) and thaflavin (Sigma, USA) on wound healing-related parameters. The first group served as the negative control (NC) and the received no treatment. The second group was designed as the vehicle control (VC) and was treated with 0.1 % dimethyl sulfodioxide (DMSO), matching the solvent concentration used in all treatment groups to rule out solvent-related effects. The third group received betanin alone at concentration of 10 μg/mL (Betn 10 μg/mL), while the fourth group was treated with theaflavin alone at 10 μg/mL (Theaf 10 μg/mL). The fifth group was treated with a combination of betanin and theaflavin, each at 10 μg/mL (Betn + Theaf 10 μg/mL) to evaluate potential synergistic effects at a lower dose. The final group received a higher combination dose of betanin and theaflavin, each at 25 μg/mL (Betn + Theaf 25 μg/mL) to assess dose - dependent responses. All treatments were applied for 24 h prior to endpoint analyses.^17^

### 2.4 MTT assay for cell viability

The effect of betanin, theaflavin, and their combinations on PDL cell viability was evaluated using the MTT assay, a colorimetric method that measures mitochondrial metabolic activity as an indicator of cell health and proliferation. Human PDL fibroblasts were seeded into 96 well culture plates at a density of cells 1 × 10^4^ cells per well and incubated for 24 h to allow for cell attachment. Following this initial incubation, the cells were subjected to the appropriate test compounds in accordance with the treatment groups that were assigned to them. Each well received 10 μL of MTT reagent (5 mg/mL in phosphate-buffered saline) after being exposed for 24 h, and the wells were then incubated for 4 h at 37 °C. Viable cells used mitochondrial dehydrogenase activity to transform the yellow tetrazolium salt into insoluble purple formazan crystals during this time. 100 μL of dimethyl sulfoxide (DMSO) was added to each well to dissolve the dormazanal crystals after the medium was carefully removed at the end of the incubation. A microplate reader was used to detect absorbance at 570 nm (Synergy, USA). By comparing the absorbance readings of the treated groups to the untreated control, the relative percentage age of cell viability was determined. All experiments were performed in triplicate to ensure statistical reliability.^18^

### 2.5 Scratch assay for cell migration

In order to simulate in vitro wound closure, the scratch test was used to evaluate the impact of betanin, theaflavin, and their combinations on the migratory capacity of human PDL fibroblasts. In six-well plates, PDL cells were planted at a density that would achieve 90–100 % confluency. A sterile 200 μL pipette tip was used to make a straight linear scratch in the middle of each well after a homogeneous monolayer had been formed. By gently washing the wells twice with phosphate buffered saline (PBS), detached cells and debris were eliminated. Cells were then subjected to the corresponding treatment groups in new culture media that included either theaflavin, betanin, or both at predetermined concentrations.^19^^,^^20^

### 2.6 Modulation of angiogenic and inflammatory gene markers

To investigate the molecular impact of betanin, theaflavin, and their combinations on wound healing processes, quantitative real time PCR (qRT-PCR) was employed to assess the expression of genes associated with angiogenesis and inflammation in PDL fibroblasts. Following a 24-h treatment period, complementary DNA (cDNA) was created by reverse transcription and total RNA was extracted using the TRIzol method. In order to assess immunomodulatory responses, the qRT-PCR was carried out using SYBR Green Master Mix and gene-specific primers for VEGF-A and HIF-1 α, which are important regulators of angiogenic signaling, as well as NF-κB and IκBα, which are essential for the regulation of inflammatory cytokines. To normalize expression data, beta-actin was employed as the internal control gene. The 2^−ΔΔCt method was used to calculate the fold changes in gene expression, and the outcomes were compared to the untreated control group. This analysis provided insight into the dual regulatory potential of the test compounds in enhancing angiogenesis and mitigating inflammation.^21^

### 2.7 Statistical analysis

All experiments were performed in triplicate (n = 3), and data were expressed as mean ± standard deviation (SD). Statistical significance was determined using Two-way analysis of variance (ANOVA) followed by Tukey's post hos test for multiple comparisons. A p-value less than 0.05 (p < 0.05) was considered statistically significant. Graphs and statistical analyses were performed using GraphPad Prism software (version 8.2).

## 3 Results

### 3.1 Cell culture and morphological observations

Human periodontal ligaments (PDL) fibroblasts were successfully cultured and maintained under standard conditions throughout the experimental period. Betanin and theaflavin were preliminary screened for their cytotoxicity using MTT assay with various range of concentrations (1–125 μg/mL; triplicate wells) as given in the Supplementary Data (Annexure. 1). From this, screening, 10 and 25 μg/mL were selected as the concentrations of interest. Both of the compounds, as individually and on combination, were subsequently tested using MTT assay.

**Fig. 1:**
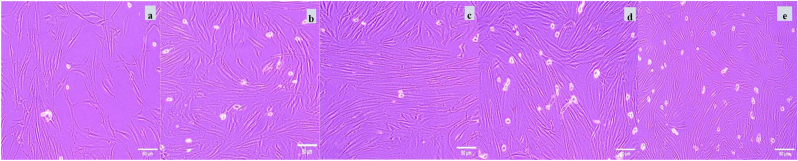
Morphological assessment of human PDL fibroblasts following treatment with betanin and theaflavin. Phase-contrast microscopic images showing the morphology of PDL fibroblasts in six treatment groups: (a) Negative control (b) Vehicle control (c)betanin (10 μg/mL), (d) theaflavin (10 μg/mL), (e)Betanin + Theaflavin (10 + 10 μg/mL), and (f) Betanin + Theaflavin (25 + 25 μg/mL). Cells in all groups retained their typical elongated, spindle-shaped morphology with no signs of cytotoxicity. Enhanced cell density and confluency were observed in the combination treatment groups, especially at 10 + 10 μg/mL, suggesting increased proliferation activity. Scale bar = 100 μm.

In the control and vehicle control groups, cells exhibited typical fibroblastic morphology. Spindle shaped with elongated cytoplasmic extensions and well-defined cell boundaries, forming a confluent monolayer. Upon treatment with betanin and theaflavin, either individually or in combination, no signs of overt cytotoxicity were observed at the tested concentrations. Cells retained their fibroblastic architecture, indicating good cytocompatibility of all formulations. Notably, in the combination treatment groups, particularly at 10 + 10 μg/mL, an increase in cell density and confluency was observed compared to the individual treatment groups, suggesting enhanced cellular proliferation. The morphological integrity and uniform distribution of cells across the culture surface further confirmed the absence of structural abnormalities, supporting the biocompatibility and potential regenerative capacity of the test compounds (Fig. 1). Based on the viability outcomes, lower, sub-cytotoxic concentrations (1 and 2.5 μg/mL) were then selected for functional assays. Specifically, these doses were used in a scratch wound migration assay to evaluate the effect of the compounds on fibroblast motility (main text, Fig. 3).

### 3.2 Assessment of metabolic activity in treated cells

**Fig. 2:**
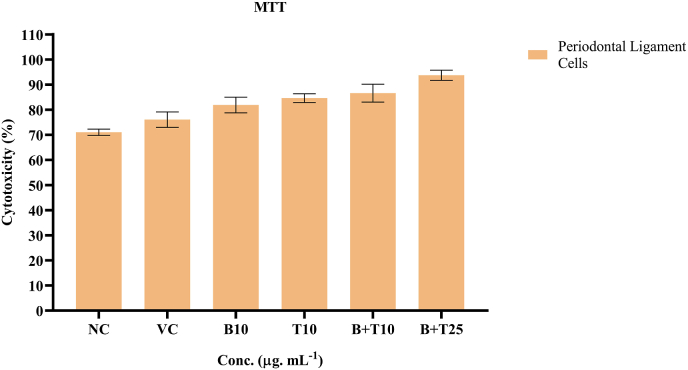
Effect of betanin theaflavin, and their combinations on PDL cell viability assessed by MTT assay. Following a 24-h treatment with Betanin (10 μg/mL), Theaflavin (10 μg/mL), and their combinations (10 + 10 μg/mL and 25 + 25 μg/mL), the percentage cell viability of human periodontal ligament (PDL) fibroblasts is shown in a bar graph in comparison to negative control (NC) and vehicle control (VC, 0.1 % DMSO). The combination at 25 + 25 μg/mL demonstrated increased cell viability comparable to the control group, suggesting good cytocompatiblility and a possible synergistic effect, whereas individual treatments showed a slight reduction in viability. Values from three separate experiments are shown as mean ± SD. Tukey's post hoc test was used after a one-way ANOVA to establish statistical significance. (∗p < 0.05).

The MTT assay results demonstrated differential effects of betanin, theaflavin, and their combinations on PDL fibroblasts viability. The negative control (NC) group exhibited high and consistent cell viability, with high values ranging from (91.61 %–94.73 %), conforming optimal baseline cell health under standard conditions. Similarly, the vehicle control (VC) group, treated with 0.1 % DMSO, showed comparable viability (89.62 %-92.71), indicating that the solvent had no cytotoxicity effect. Treatment with betanin alone at 10 μg/mL resulted in a modest reduction in viability (78.59 %–84.74 %), suggesting mild cytotoxic stress or reduced proliferative capacity at this concentration. Theaflavin alone at 10 μg/mL showed slightly higher viability (83.6 %–86.6 %) compared to betanin, indicating a more favorable cyto-compatibility profile (Fig. 2).

**Fig. 3:**
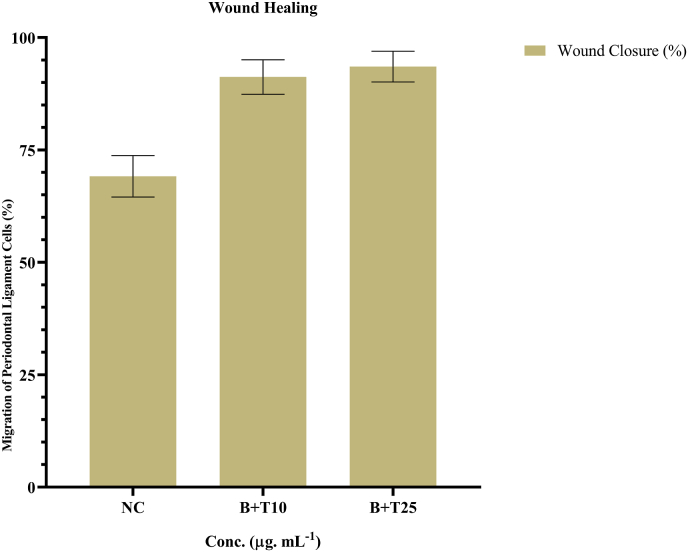
Effect of betanin and theaflavin combination on PDL fibroblast migration assessed by scratch assay. Bar graph representing the percentage of wound closure after 24 h of treatment with betanin + theaflavin at 1 μg/mL concentrations, compared to the negative control (NC). Doses were selected based on IC_50_ analysis to ensure sub-cytotoxic exposure. Cells treated with the combination showed significantly enhanced migration, with the 2.5 μg/mL group achieving the highest closure percentage, indicating strong pro-migratory potential. Data are expressed as mean ± SD from three independent experiments. Statistical analysis was performed using one-way ANOVA followed by Tukey's post hoc test (∗p < 0.05).

Inverted microscopic images showing the morphology of PDL fibroblasts in six treatment groups: (a) Negative control (b) Vehicle control (c)betanin (10 μg/mL), (d) theaflavin (10 μg/mL), (e)Betanin + Theaflavin (10 + 10 μg/mL), and (f) Betanin + Theaflavin (25 + 25 μg/mL). Cells in all groups retained their typical elongated, spindle-shaped morphology with no signs of cytotoxicity. Enhanced cell density and confluency were observed in the combination treatment groups, especially at 10 + 10 μg/mL, suggesting increased proliferation activity. Scale bar = 100 μm.

Following a 24-h treatment with Betanin (10 μg/mL), Theaflavin (10 μg/mL), and their combinations (10 + 10 μg/mL and 25 + 25 μg/mL), the percentage cell viability of human periodontal ligament (PDL) fibroblasts is shown in a bar graph in comparison to negative control (NC) and vehicle control (VC, 0.1 % DMSO). The combination at 25 + 25 μg/mL demonstrated increased cell viability comparable to the control group, suggesting good cytocompatiblility and a possible synergistic effect, whereas individual treatments showed a slight reduction in viability. Values from three separate experiments are shown as mean ± SD. Tukey's post hoc test was used after a Two-way ANOVA to establish statistical significance. (∗p < 0.05, (F (1, 20) = 436.4, p < 0.0001) and concentration (F (4, 20) = 20.75, p < 0.0001), as well as a significant interaction (F (4, 20) = 12.41, p < 0.0001). Post-hoc analysis revealed that betanin and theaflavin at 2.5 μg/mL had mean increase of 2.24 units as comparable with negative control (NC (95 % CI: 2.467 to −2.019, p < 0.0001). These results showed that the statistical effective size and its significance.

### 3.3 Evaluation of fibroblast migration following combination treatment

**Fig. 4:**
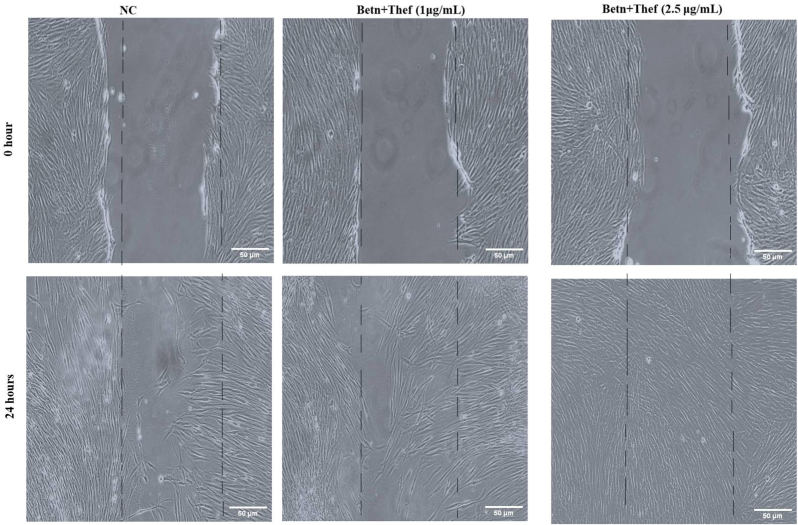
Representative Scratch assay images showing wound closure in PDL fibroblasts after 24 h of treatment. Images display untreated control and cells treated with betanin + theaflavin at 1 μg/mL and 2.5 μg/mL. Control group shows partial wound closure, while treatment groups exhibit enhanced cell migration, with the 2.5 μg/mL group showing nearly complete closure of the scratch area, indicating strong pro-healing effects of the combination treatment. Scale bar = 100 μg/mL.

Scratch assay results demonstrated a dose-dependent enhancement in the migratory capacity of PDL fibroblasts treated with the combination of betanin and theaflavin (Fig. 3). Based on preliminary MTT analysis and IC calculations, two sub-cytotoxic concentrations, 1 μg/mL and 2.5 μg/mL, were selected for evaluating wound closure. After 24 h of treatment, the combination-treated groups exhibited significantly greater wound closure of approximately 69 %, while the 1 μg/mL combination group showed an average closure rates ranging from 75.56 % to 78.96 %. Notability the 2.5 μg/mL combination group demonstrated superior closure efficiency, with wound closure values between 91.34 % and 97.47 %, indicating enhanced migratory activity. These findings suggest that the combined application of betanin and theaflavin at optimized low doses significantly promotes cell migration and in vitro wound healing in a concentration-dependent manner (Fig. 4). The statistical significance was evaluated with the One-way ANOVA (F (2,6) = 43.8, p < 0.0001). The post-hoc comparison (Tukey's test) indicated that Betn + Theaf at 1 μg/mL significantly increased values compared with NC (mean difference 8.42, 95 % CI: 2.23–14.6, p < 0.01). The 2.5 μg/mL dose produced an even greater increase compared with NC (mean difference 24.40, 95 % CI: 18.2–30.6, p < 0.0001), and also differed significantly from the 1 μg/mL group (mean difference 15.98, 95 % CI: 9.8–22.2, p < 0.001). These results confirm a clear dose-dependent enhancement.

Bar graph representing the percentage of wound closure after 24 h of treatment with betanin + theaflavin at 1 μg/mL concentrations, compared to the negative control (NC). Doses were selected based on IC_50_ analysis to ensure sub-cytotoxic exposure. Cells treated with the combination showed significantly enhanced migration, with the 2.5 μg/mL group achieving the highest closure percentage, indicating strong pro-migratory potential. Data are expressed as mean ± SD from three independent experiments. Statistical analysis was performed using one-way ANOVA followed by Tukey's post hoc test (∗p < 0.05).

### 3.4 Modulation of angiogenic and inflammatory gene expression

**Fig. 5:**
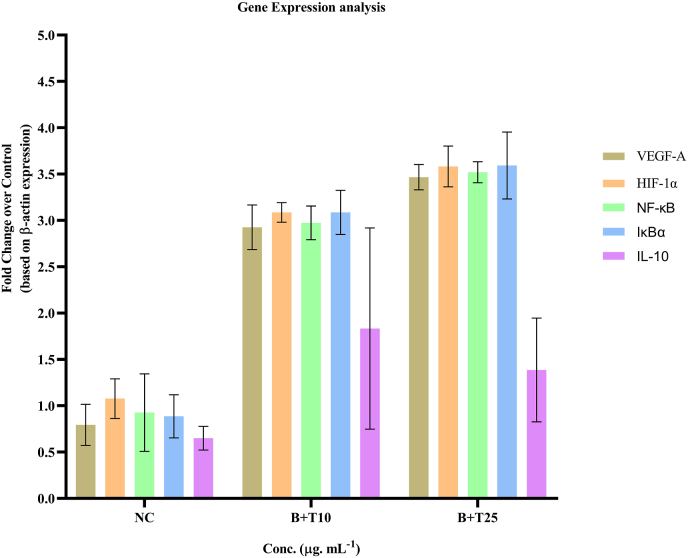
Relative gene expression levels of VEGF-A, HIFα, NF-κB, IκBα and IL-10 in PDL fibroblasts following treatment with betanin and theaflavin (2.5 μg/mL each). Data are presented as fold changes compared to untreated control, normalized to β-actin. Upregulation of VEGF-A and HIFα indicates enhanced angiogenic signaling, while downregulation of NF-κB and concurrent increases in IκBα and IL-10 reflect anti-inflammatory and regulatory effects. Values represent mean ± SD from three independent experiments. Statistical significance was determined using one-way ANOVA followed by Tukey's post hoc test (∗p < 0.05).

Gene expression analysis revealed significant molecular changes in PDL fibroblasts following treatment with the concentration of betanin and theaflavin. At 2.5 μg/mL, the combination treatment resulted in marked upregulation of angiogenic markers VEGF-A and HIFα, indicating enhanced potential for neovascularization and tissue regeneration. Concurrently, there was a notable downregulation of NF-κB, a key transcription factor associated with pro-inflammatory signaling. This reduction was accompanied by increased expression of IκBα, The natural inhibitor of NF-κB, suggesting suppression of inflammatory responses at the transcriptional level. Additionally, a significant increase in the expression of IL-10, an anti-inflammatory cytokine, was observed, supporting the immunomodulatory capacity of the phytochemical combination. These results confirm that the co-treatment not only promotes pro-healing angiogenic pathways but also attenuates inflammation, thereby establishing a favorable environment for wound repair (Fig. 5). Data are presented as fold changes compared to untreated control, normalized to β-actin. Upregulation of VEGF-A and HIFα indicates enhanced angiogenic signaling, while downregulation of NF-κB and concurrent increases in IκBα and IL-10 reflect anti-inflammatory and regulatory effects. Values represent mean ± SD from three independent experiments. Statistical significance was determined using Two-way ANOVA followed by Tukey's post hoc test (∗p < 0.05).

## 4 Discussion

Wound healing is a vital physiological process that requires the coordinated regulation of cellular proliferation, migration, angiogenesis, and inflammation resolution. Disruption in any of these processes may result in delayed healing or chronic wounds. Recent research has increasingly turned to natural compounds with multitargeted actions for their potential to modulate these processes with minimal cytotoxicity. Tn this study, we explored the in vitro effects of betanin and theaflavin, two plant-derived bioactives, on human periodontal ligament (PDL) fibroblasts, focusing on their influence on cell viability, migration, and the expression of angiogenic and inflammatory genes. Our findings suggest that the combination of betanin and theaflavin significantly enhances cellular responses associated with wound repair.^22^

The MTT assay results demonstrated that the combination of betanin and theaflavin at 25 + 25 μg/mL promoted cell viability comparable to the untreated control. Interestingly, individual treatments with betanin or theaflavin at 10 μg/mL exhibited a moderate reduction in cell viability, which may reflect a threshold effect where lower concentrations alone are insufficient to stimulate cellular proliferation. However, the combination treatment restored and even slightly enhanced viability, indicating a possible synergistic interaction. This observation aligns with the hypothesis that combining compounds with complementary bioactivities can migrate individual limitations and amplify overall therapeutic efficacy.^23^

The enhanced cell viability observed under combination of treatment may be attributed to the antioxidant properties of both betanin and theaflavin. Reactive oxygen species (ROS) accumulation at the wound site is known to impair cell proliferation and survival. By scavenging ROS, these compounds may create a more favorable intracellular environment, supporting fibroblast function and proliferation. Moreover, antioxidant-mediated protection of mitochondrial function could explain higher metabolic activity observed in the MTT assay.^24^

In terms of cell migration, a key event during the proliferative phase of wound healing, the scratch assay provided critical insights. At lower concentrations of 1 μg/mL and 2.5 μg/mL determined based on prior IC evaluations, the combination of betanin and theaflavin significantly enhanced wound closure in PDL fibroblasts compared to the control group. Notably, the 2.5 μg/mL combination group showed the highest migration rate, suggesting a dose-response effect. These findings support the idea that even sub-cytotoxic doses of natural compounds can stimulate fibroblast migration, possibly through modulation of cytoskeletal dynamics and extracellular matrix remodeling. Enhanced migratory behavior is essential for timely wound closure and tissue regeneration, especially in oral tissues where rapid repair is required to maintain barrier function.

We examined the expression of genes related to inflammation and angiogenesis in two highly regulated pathways in wound repair in order to learn more about the underlying biological mechanisms. The combination of betanin and theaflavin at 2.5 μg/mL led to significant upregulation of VEGF-A and HIFα, both of which are central regulators of neovascularization. VEGF-A is known to stimulate endothelial cell proliferation and capillary formation, while HIFα acts as a transcriptional activator under hypoxic conditions, commonly present in the early wound microenvironment. The upregulation of these markers implies that the combination treatment may enhance angiogenic signaling, facilitating nutrient and oxygen delivery necessary for sustained tissue regeneration.^25^

Concurrently, gene expression analysis revealed a notable downregulation of NF-κB, a pro-inflammatory transcription factor often associated with chronic inflammation and impaired healing. The reduced expression of NF-κB suggests suppression of inflammatory signaling pathways, which, if left unchecked, could lead to prolonged tissue damage. Additionally, the observed upregulation of IκBα, a cytoplasmic inhibitor of NF-κB, further confirms the anti-inflammatory effect of the combination. By preventing NF-κB nuclear translocation, IκBα plays a protective role in limiting the expression of downstream inflammatory mediators such as IL-6, TNF-α, AND COX-2. Crucially, the phytochemical combination's immunoregulatory potential is shown by the notable elevation of IL-10, a strong anti-inflammatory cytokine. In addition to limiting leukocyte infiltration and promoting tissue homeostasis, IL-10 inhibits the expression of pro-inflammatory cytokines. The concurrent downregulation of NF-κB and upregulation of IL-10 suggest that betanin and theaflavin do not merely suppress inflammation but actively promote its resolution, which is critical aspect of transitioning from the inflammatory to the proliferative phase in wound healing.^26^

The integration of these cellular and molecular outcomes supports a dual-action model for betanin and theaflavin in wound healing: they enhance tissue regeneration by simultaneously promoting angiogenesis and suppressing detrimental inflammatory responses. This synergistic approach is particularly valuable given that chronic wounds often results from the coexistence of vascular insufficiency and unresolved inflammation. Most conventional therapies target either one or the other, often leading to incomplete or temporary healing. In contrast, a dual-pathway strategy may offer a more comprehensive solution.

The use of human PDL fibroblasts in this study adds translational value, as these cells are directly involved in the maintenance and repair of periodontal structures. Enhancing their viability and function could have significant implications for periodontal wound healing and regenerative dentistry. Moreover, the use of low dose combinations of natural compounds reduces the risk of cytotoxicity and supports their potential application in topical formulations, wound dressings, or biomaterial coatings. Despite the study's encouraging results, a number of caveats must be noted. Initially, this study was restricted to in vitro settings, which are unable to accurately mimic the intricacy of in vivo wound environments, such as vascular networks, extracellular matrix remodeling, and immune cell interactions. Future studies should incorporate animal models or 3D co-culture systems to validate the in vivo efficacy and safety of the combination. Second, mechanistic studies exploring upstream regulators and downstream targets of VEGF-A, HIFα, and NF-κB pathways would provide deeper insights into the precise signaling cascades affected by these bioactives.^27^^,^^28^

Another consideration is the pharmacokinetics and bioavailability of betanin and theaflavin when applied topically or systemically. While both compounds are known for their biological activity, their stability in biological fluids and permeability through skin or mucosal barriers will influence their clinical effectiveness. Encapsulation techniques such as nanoparticle carriers or hydrogel matrices may enhance delivery and retention at the wound site. Despite these limitations, this study provides strong preliminary evidence supporting the use of a betanin and theaflavin combinations as a multifunctional wound healing agent. Their dual modulation profiles, underscores and inflammation, coupled with favorable cell viability and migration profiles, underscores their potential for further development into therapeutic formulations aimed at managing both acute and chronic wounds.^29^ On further validation, these nanoparticles would help in reducing the healing time, infection risk, and patient's comfort on routine applications and surgeries.

## 5 Conclusion

This in vitro study demonstrated that the combined application of betanin and theaflavin significantly enhances key cellular and molecular processes involved in wound healing. Along with increasing PDL fibroblast movement and viability, the combination significantly changed the expression of important genes linked to inflammation and angiogenesis. While downregulation of NF-κB and overexpression of IκBα and IL-10 emphasize the substances' anti-inflammatory and immunoregulatory properties, elevation of VEGF-A and HIFα promotes increased neovascularization. These findings suggest a synergistic interaction between the two phytochemicals, offering a promising dual action strategy for promoting tissue repair. Further in vivo validation and formulation development are warranted to translate these findings into clinical applications for the management of oral, dermal, and chronic wounds.

## Patient's/Guardian's consent

The authors declare that the above-mentioned manuscript has no need of Patient's/Guardian's consent since, it is a non-clinical research manuscript.

## Declaration of ethical clearance

This study was conducted using only in vitro cell culture methods. No human or animal subjects were involved, so ethical clearance was not required.

## Declaration of competing interest

The authors declare that they have no known competing financial interests or personal relationships that could have appeared to influence the work reported in this paper.
