# Supplementary material for: Dual pathway activation in wound repair: An in vitro study of betanin and theaflavin on periodontal ligament fibroblasts
Source: J Oral Biol Craniofac Res. 2025 Nov 11;16(1):45–51. doi: 10.1016/j.jobcr.2025.10.020 (PMC12648945; doi:10.1016/j.jobcr.2025.10.020)
Supplement: Multimedia component 1 [file mmc1.docx]

**Annexure I**

The eight concentrations (*viz.,* 1, 5, 10, 25, 50, 75, 100, 125 µg/mL) were tested for their cytotoxic activity on periodontal ligament cells using MTT assay (Figure.S1). Doxorubicin was used as the positive control. The results showed that the betanin and theaflavin were showed lesser toxicity than doxorubicin. IC_50_ was calculated as 110 and 130 µg/mL, for betanin and theaflavin, respectively. Statistically, Two way ANOVA showed that significance between the concentrations (F(7,48) = 195.6, p < 0.0001) and set (F(2,48) = 3032, p < 0.0001), also, a significant concentration × set interaction (F(14,48) = 26.79, p < 0.0001). Post hoc Tukey’s multiple comparisons confirmed that Set A consistently exhibited significantly lower viability than Sets B and C at concentrations ≥5 µg/mL (p < 0.01). The treatment concentrations such as 10 and 25 µg/mL were selected and further tested with the PDL cells using MTT assay. The viability (approximately, 90%) was confirmed for the execution of the migration assay at the specified concentrations for both of the compounds.


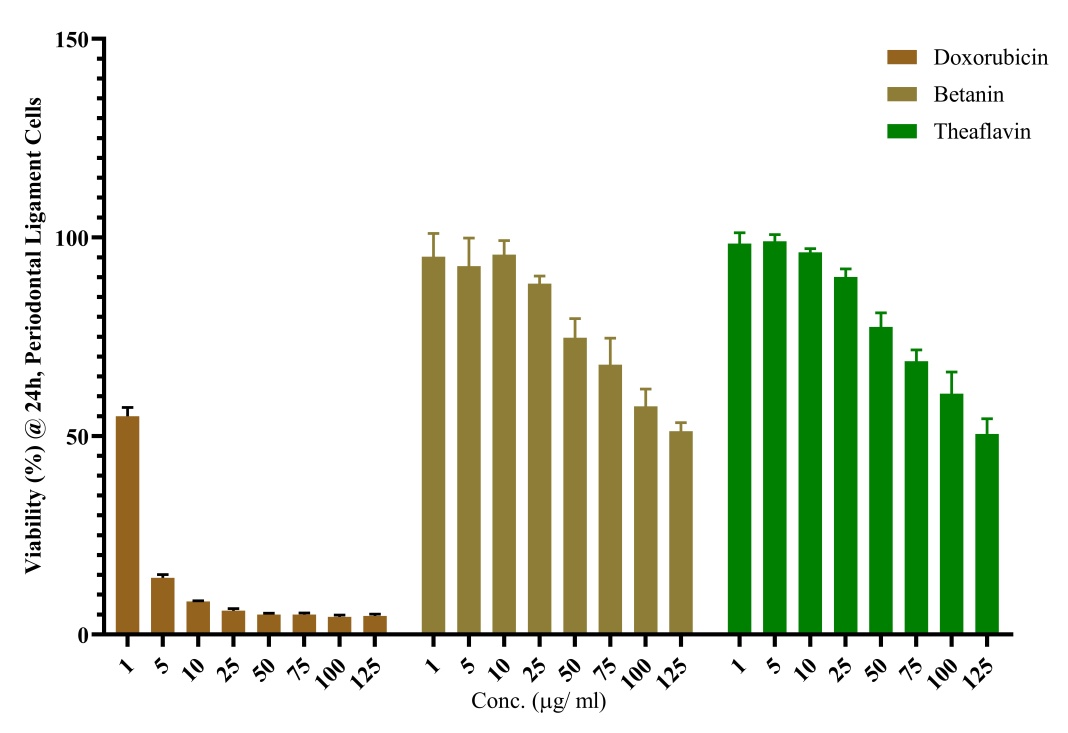


**Figure S1. MTT assay showing the dose-dependent cytotoxic effect of doxorubicin, betanin and theaflavin on periodontal ligament cells (PDL cells).** Increasing concentrations (1–125 µg/mL) resulted in a progressive reduction in cell viability, with IC₅₀ estimated at 8, 110 and 130 µg/m, as respectively. Data represent mean ± SD of three independent experiments performed in triplicate (n=3).
